# KSR induces RAS‐independent MAPK pathway activation and modulates the efficacy of KRAS inhibitors

**DOI:** 10.1002/1878-0261.13213

**Published:** 2022-04-12

**Authors:** Guillem Paniagua, Harrys K.C. Jacob, Oksana Brehey, Sara García‐Alonso, Carmen G. Lechuga, Tirso Pons, Monica Musteanu, Carmen Guerra, Matthias Drosten, Mariano Barbacid

**Affiliations:** ^1^ Experimental Oncology Molecular Oncology Program Centro Nacional de Investigaciones Oncológicas (CNIO) Madrid Spain; ^2^ Department of Immunology and Oncology National Center for Biotechnology (CNB‐CSIC) Spanish National Research Council Madrid Spain; ^3^ Department of Biochemistry and Molecular Biology Faculty of Pharmacy Complutense University Madrid Spain; ^4^ Present address: Department of Surgery Sylvester Comprehensive Cancer Center University of Miami FL USA; ^5^ Present address: Molecular Mechanisms of Cancer Program Centro de Investigación del Cáncer and Instituto de Biología Molecular y Celular del Cáncer CSIC‐University of Salamanca Spain

**Keywords:** ATP binding, KRAS^G12C^, MAPK pathway, RAS‐independent proliferation, resistance, sotorasib

## Abstract

The kinase suppressor of rat sarcoma (RAS) proteins (KSR1 and KSR2) have long been considered as scaffolding proteins required for optimal mitogen‐activated protein kinase (MAPK) pathway signalling. However, recent evidence suggests that they play a more complex role within this pathway. Here, we demonstrate that ectopic expression of KSR1 or KSR2 is sufficient to activate the MAPK pathway and to induce cell proliferation in the absence of RAS proteins. In contrast, the ectopic expression of KSR proteins is not sufficient to induce cell proliferation in the absence of either rapidly accelerated fibrosarcoma (RAF) or MAPK‐ERK kinase proteins, indicating that they act upstream of RAF. Indeed, KSR1 requires dimerization with at least one member of the RAF family to stimulate proliferation, an event that results in the translocation of the heterodimerized RAF protein to the cell membrane. Mutations in the conserved aspartic acid–phenylalanine–glycine motif of KSR1 that affect ATP binding impair the induction of cell proliferation. We also show that increased expression levels of KSR1 decrease the responsiveness to the KRAS^G12C^ inhibitor sotorasib in human cancer cell lines, thus suggesting that increased levels of expression of KSR may make tumour cells less dependent on KRAS oncogenic signalling.

## Graphical abstract

Ectopic expression of kinase suppressor of rat sarcoma (RAS) 1 or 2 (KSR1 or KSR2) induces cell proliferation in the absence of RAS. RAS‐independent proliferation occurs via mitogen‐activated protein kinase (MAPK) pathway activation through the heterodimerization and activation of rapidly accelerated fibrosarcoma (RAF) proteins, an activity that requires their plasma membrane localization and ATP binding. Increased expression levels of KSR make tumor cells less dependent on KRAS signaling and thus possibly less responsive to KRAS inhibitors.

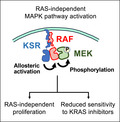

**Abbreviations**
CA: conserved area
DFG: aspartic acid–phenylalanine–glycine
ERK: extracellular signal‐regulated protein kinase
HRD: histidine–arginine–aspartic acid
KSR: kinase suppressor of RAS
MAPK: mitogen‐activated protein kinase
MEK: MAPK‐ERK kinase
PDX: patient‐derived xenograft
RAF: rapidly accelerated fibrosarcoma
RAS: rat sarcoma

## 1 Introduction

The kinase suppressor of rat sarcoma (RAS) proteins (KSR), originally identified in *Drosophila* and *Caenorhabditis elegans*, have gained special interest due to their requirement for optimal mitogen‐activated protein kinase (MAPK) pathway activation [1, 2]. Mammals harbour two KSR paralogs, KSR1 and KSR2, thought to act as scaffolds for the MAPK pathway. Mice lacking *Ksr1* are phenotypically normal, although optimal MAPK pathway activation and transformation by RAS oncogenes is attenuated [3, 4]. In contrast, one third of *Ksr2*‐deficient mice display perinatal mortality and the surviving animals develop obesity and insulin‐resistance [5]. Consistent with a selective role of KSR2 in controlling energy expenditure, *KSR2* mutations associated with obesity and insulin resistance have been found in humans [6]. This activity has been attributed to an extra motif not present in KSR1 that mediates binding to 5′‐AMP‐activated protein kinase. In addition, KSR2 also connects calcium signalling to MAPK activation via these extra sequences [5, 7].

Biochemical studies have shown that KSR proteins interact with rapidly accelerated fibrosarcoma (RAF), MAPK‐ERK kinase (MEK) and extracellular signal‐regulated protein kinase (ERK) and facilitate ERK activation in a dose‐dependent manner [8, 9]. Under resting conditions, KSR proteins sequester MEK in the cytoplasm [10]. Upon RAS activation, a series of dephosphorylation events within KSR disable the inhibitory association with 14‐3‐3 and promote its membrane relocalization to foster MEK phosphorylation by RAF kinases [10, 11, 12]. However, only when IMP (also known as BRAP), an E3 ligase that binds the inactive KSR/MEK complex and contributes to its cytoplasmic sequestration, displaces from KSR to active RAS, KSR can fully translocate to the plasma membrane [13, 14].

Kinase suppressor of RAS proteins share a substantial degree of homology with RAF kinases and have been shown to form heterodimers with RAF proteins [15]. However, there has been much debate as to whether KSR proteins also function as protein kinases. Its kinase domain lacks several key amino acids believed to be required for the phosphotransfer reaction, which resulted in its classification as a pseudokinase [16, 17, 18]. Yet, a series of studies have detected ATP‐binding and kinase activity in *in vitro* assays, suggesting that KSR may indeed possess at least some low levels of kinase activity [19, 20, 21, 22]. A model based on the structure of the KSR2 kinase domain proposed that BRAF, when bound to KSR2, can allosterically activate the putative kinase activity of KSR2, which results in the phosphorylation of the N‐terminal domain of MEK1. This model also predicts the involvement of a second BRAF molecule responsible for the full activation of MEK1 [22]. Recently, a different model has been proposed in which MEK binds to KSR inducing a conformational change that allows it to heterodimerize with BRAF. These KSR‐BRAF heterodimers are then responsible for phosphorylating and activating MEK [23, 24]. This model is consistent with a previous report indicating that KSR proteins stimulate RAF activation in a kinase‐independent manner [25].

Based on these observations, we reasoned that KSR proteins may, at least under certain conditions, promote the activation of the MAPK pathway in a RAS‐independent manner, as previously described for RAF proteins [26]. Here, we show that increased expression levels of KSR proteins induce the proliferation of MEFs devoid of RAS proteins by a mechanism involving membrane recruitment of RAF proteins via heterodimerization as well as ATP‐binding. As a consequence, KSR overexpression reduces the dependency of cancer cells on KRAS signalling, limiting the efficacy of KRAS inhibitors.

## 2 Materials and methods

### 2.1 Cell culture and treatments

*Hras* ^–/–^;*Nras* ^–/–^;*Kras* ^lox/lox^;RERT^ert/ert^ (*Kras*lox) MEFs were established in our laboratory and colony assays in RASless cells have been described previously [26]. Briefly, cells stably expressing retroviral or lentiviral vectors were seeded at equal cell numbers in the absence or presence of 600 nm 4‐Hydroxytamoxifen (4OHT). Colonies were stained with crystal violet after 2 weeks. *Araf* ^lox/lox^;*Braf* ^lox/lox^;*Raf1* ^lox/lox^ (*Raf*lox) MEFs have also been published [27]. *Raf*lox cells stably expressing retroviral or lentiviral vectors were infected with Adeno‐GFP or Adeno‐Cre (moi 100) and equal cell numbers were seeded for colony formation 3 days later. In all colony assays, untreated or Adeno‐GFP‐infected plates had at least 100 colonies. Doxorubicin (Sigma‐Aldrich, Gillingham, Dorset, UK) was used at a final concentration of 5 μg·mL^−1^ for 16 h. Human MIA PaCa‐2 tumour cells were obtained from ATCC, Manassas, VA, USA. NIH3T3 cells were obtained from Stuart Aaronson [28]. Patient‐derived xenograft (PDX)‐derived cell lines PDX‐dc1 and PDX‐dc2 were established in our laboratory from PDX tumours and have been described previously [29]. Sotorasib (AMG 510) was purchased from MedChemExpress, Monmouth Junction, NJ, USA and was used at the indicated concentrations.

### 2.2 Plasmids and viral vector production

Retroviral or lentiviral vectors were produced as described in 293T cells using the pCL‐Eco packaging vector system or the ViraPower Lentiviral packaging mix (Thermo Fisher Scientific, Waltham, MA, USA), respectively [30]. pMSCV‐KSR1‐IRES‐GFP (Addgene #25973; Watertown, MA, USA) and pMSCV‐KSR2‐IRES‐GFP (Addgene #25969) were a gift from Rob Lewis [9]. KSR1 deletion mutants were generated by PCR using a forward primer specific for the corresponding position in KSR1 that included a sequence encoding an N‐terminal V5 tag and a common reverse primer. PCR products were subcloned using the pCR8/GW/Topo TA Cloning kit (Invitrogen, Renfrew, UK) following the manufacturer's instructions and sequence‐verified. Constructs expressing the CA1 or CA1–4 were generated by the introduction of a stop codon after the respective domains into the wild‐type V5‐KSR1 cDNA. All deletion mutants were subsequently cloned into pLentiCMVPuroDEST (Addgene #17452) and pLentiCMVHygroDEST (Addgene #17454) using the Gateway LR Clonase II enzyme mix (Invitrogen) following the guidelines of the manufacturer. Mutations in KSR1 proteins (D683A, D700A, D701F, R732H, R615H) were introduced into pMSCV‐KSR1‐IRES‐GFP using the QuikChange Lightning site‐directed mutagenesis kit (Agilent, Santa Clara, CA, USA). Likewise, the R509H or the D594A mutation were introduced into pBABEhygro BRAF, whereas the R401H or D468A mutation were introduced into pBABEpuro RAF1 [26]. The KSR1caax construct was generated by the addition of the CAAX box sequence including the polybasic stretch of KRAS to the C‐terminus of KSR1 by PCR. The resulting KSR1caax cDNA was subsequently cloned into pMSCV‐IRES‐GFP. Viral vectors expressing KRAS^G12V^ and sh‐*p53* have been described [26, 27]. To purify recombinant mouse KSR1 as well as KSR1^F701A^, KSR1^D683A^, KSR1^D700A^ or KSR1^A598F^, the corresponding cDNAs were amplified by PCR with restriction site‐tailed primers incorporating a C‐terminal StrepTagII separated by a Gly‐Ser‐Ala linker. The resulting PCR product was cloned into the HindIII/XhoI sites of pcDNA3 (Invitrogen) using the In‐Fusion HD EcoDry Cloning Kit (Takara Bio, Shiga, Japan). The cDNA clone for full‐length human CDC37 tagged with a C‐terminal Myc‐DDK tag was purchased from Origene (Rockville, MD, USA; RC201002). SgRNAs targeting *Ksr1* (#1, TTGGCGTGCCGTCGTAGCGT; #2, CGATGGGCGAGAAAAAGGAG; #3, GCAGGAGATCCGGACCCTAG) were cloned into lentiCRISPRv2 (Addgene 52961). The sgRNA targeting *p53* (GTGTAATAGCTCCTGCATGG) was cloned into lentiCRISPRv1 (Addgene #49535) after replacing the sequences encoding the puromycin resistance gene with those of the blasticidin resistance gene.

### 2.3 Protein analysis

For western blot analysis, cells were lysed in 50 mm Tris‐HCl (pH 7.4), 150 mm NaCl, 0.5% NP‐40 supplemented with the Complete Mini protease inhibitor cocktail (Roche) and phosphatase inhibitor cocktails 2 and 3 (Sigma). Usually, 25 μg total protein lysate were separated by SDS/PAGE and transferred to nitrocellulose membranes. Antibodies raised against the following proteins were used: KSR1 (BD Biosciences, San Jose, CA, USA, 611576; Santa Cruz Biotechnology, Dallas, TX, USA, 25416 and Abcam, Waltham, MA, USA, ab68483), KSR2 (Abnova, Taipei City, Taiwan, H00283455‐M08), ARAF (Cell Signaling, 4432), BRAF (Santa Cruz, sc‐5284), RAF1 (BD Biosciences, 610151), MEK1 (Santa Cruz, sc‐219), MEK2 (Santa Cruz, sc‐524), pMEK1/2 (Cell Signaling Technology, Danvers, MA, USA, 9154), ERK1 (BD Pharmingen, San Diego, CA, USA, 554100), ERK2 (BD Biosciences, 610103), pERK1/2 (Cell Signaling, 9101), pan RAS (Calbiochem, San Diego, CA, USA, OP40), RHOGDI (Cell Signaling, 2564), ITGB1 (Cell Signaling, 34971), V5‐tag (Invitrogen, R96025), FLAG‐M2 (Sigma, F3165), p53 (Cell Signaling, 2524), GAPDH (Sigma, G8795) and Vinculin (Sigma, V9131). For immunoprecipitations, 2 mg of total protein lysate were incubated with 1 μg primary antibody on a rotating wheel for 2 h at 4 °C. To precipitate antibody/protein complexes, TrueBlot IP beads (Rockland, Pottstown, PA, USA) were washed three times in 50 mm Tris‐HCl (pH 7.4), 150 mm NaCl, 0.5% NP‐40 buffer and incubated with the protein lysates overnight at 4 °C on a rotating wheel. The next day, beads were washed three times in 50 mm Tris‐HCl (pH 7.4), 150 mm NaCl, 0.5% NP‐40 buffer followed by three additional times in PBS. Samples were then eluted by resuspending the beads in SDS sample loading buffer and boiling for 10 min, followed by SDS/PAGE. For the immunoprecipitation of proteins carrying the V5‐tag, we used the V5‐Trap agarose (ChromoTek, Planegg‐Martinsried, Germany) following guidelines of the manufacturer.

### 2.4 Recombinant protein purification, kinase assay and ATP binding

To purify recombinant KSR proteins, Expi293F cells (Thermo Fisher Scientific) were maintained in Expi293F Expression Medium (Thermo Fisher Scientific) and expanded following the recommendations of the supplier. The day before transfection, cells were seeded at 2 × 10^6^/mL and allowed to grow overnight. For each transfection, cells were diluted to a final density of 3 × 10^6^/mL in a total volume of 120 mL (in a 500 mL flask), with fresh prewarmed Expi293F Expression Medium. A total of 120 µg plasmid DNA (88 µg pcDNA3‐KSR1*‐Strep + 32 µg pCMV6‐CDC37‐Myc‐DDK) was diluted with 6 mL OptiMEM I‐GlutaMAX (Thermo Fisher Scientific) and mixed by inversion. Similarly, 480 µL of PEI reagent (1 mg·mL^−1^) was diluted with 6 mL OptiMEM® and gently mixed by inversion (Thermo Fisher Scientific). The mixture was added and cells were harvested after 48 h by resuspending them in 15 mL lysis buffer (20 mm Tris‐HCl [pH 7.5], 150 mm NaCl, 10 mm MgCl_2_, 10 mm KCl, 20 mm Na_2_MoO_4_ and 0.1% Triton X‐100) supplemented with Complete Mini Protease Inhibitor Cocktail (Merck, Darmstadt, Germany) and Phosphatase Inhibitor Cocktails 2 and 3 (Sigma) and agitation at 4 °C for 15 min. After a brief sonication, the lysate was centrifuged (21 000 *g* 20 min, 4 °C) and the soluble fraction was purified by affinity chromatography using a 5‐mL StrepTrap column (GE Healthcare, Wauwatosa, WI, USA), on an ÄKTA prime (GE Healthcare). The StrepTrap column was washed with 20 column volumes of buffer A1 [20 mm Tris‐HCl (pH 7.5), 150 mm NaCl, 10 mm MgCl_2_, 10 mm KCl, 20 mm Na_2_MoO_4_] and eluted with a stepwise gradient (0–100%) of buffer B1 [20 mm Tris‐HCl (pH 7.5), 150 mm NaCl, 10 mm MgCl_2_, 10 mm KCl, 20 mm Na_2_MoO_4_ and 2.5 mm desthiobiotin]. KSR1*‐containing fractions were pooled, concentrated with a 30‐kDa Vivaspin concentrator (Sartorius, Göttingen, Germany) to final concentration of about 1 mg·mL^−1^, and stored at −80 °C until use. For kinase assays, 2 μg of the purified recombinant KSR1 as well as KSR1^F701A^ proteins were incubated with 10 μCi γ‐^32^P‐ATP in kinase buffer (25 mm Hepes pH 7.5, 5 mm MgCl_2_, 2.5 mm MnCl_2_, 1 mm DTT, 0.1 mm Na_3_VO_4_, 1 mm ATP) for 30 min at 30 °C prior to separation by SDS‐PAGE. As a positive control, we used recombinant active RAF1 (Sigma, 14‐352) and as a substrate recombinant MEK1 (Sigma, 14‐420). To measure ATP binding by KSR1 proteins, 2 μg of the purified recombinant KSR1, KSR1^A587F^ as well as KSR1^F701A^ proteins were incubated with 10 μCi γ‐^32^P‐ATP in binding buffer (20 mm Na_2_HPO_4_/NaH_2_PO_4_ pH 7.2, 10 mm MgCl_2_) for 5 min on ice. The specificity was confirmed by competing with 5 μm unlabeled (cold) ATP. Samples were cross‐linked by UV exposure for 2 min prior to separation by SDS/PAGE.

### 2.5 Membrane fractionation

The plasma membrane fractions were separated using the Plasma Membrane Protein Extraction Kit (Abcam) following instructions of the manufacturer.

### 2.6 Immunofluorescence staining

Cells were seeded on glass coverslips, fixed with 4% paraformaldehyde and permeabilized with 0.5% Triton X‐100 for 5 min. Coverslips were blocked with 3% BSA in PBS for 45 min and incubated with anti‐V5 antibodies (Invitrogen; R960‐25) in PBS with 3% BSA for 1 h at 37 °C. After washing three times with PBS for 5 min, coverslips were incubated with goat anti‐mouse Alexa Fluor 594 secondary antibodies for 1 h in PBS with 3% BSA. Finally, coverslips were washed again three times with PBS for 5 min and nuclei were counterstained with Hoechst 33342. Cells were imaged using a Leica Biosystems (Nussloch, Germany) TCS SP5 confocal microscope.

### 2.7 T7 endonuclease assay

A PCR was performed on genomic DNA extracted from cells infected with *Ksr1* or scrambled sgRNAs. For sgRNA #1, the following primer combination was used (F, TCACGGTGTCATGCTCT and R, TGATACACGGCACCATT). For sgRNAs #2 and #3, the following primer combination was used (F, GCCAGAAAGCAGATGCGA and R, TCACGCTGCCGCATCAG). Each PCR product (200 ng) was incubated for 10 min at 95 °C, slowly cooled down to 30 °C and incubated for 1 h with 10 units T7 endonuclease (New England Biolabs, Ipswich, MA, USA) at 37 °C. Samples were separated on a 10% polyacrylamide gel in TBE buffer. For *Ksr1* sgRNA #1, the expected size of the PCR product was 513 bp and cleavage by Cas9 resulted in fragments of 362 and 151 bp. For *Ksr1* sgRNA #2, the expected size of the PCR product was 547 bp and cleavage by Cas9 resulted in fragments of 338 and 209 bp. For *Ksr1* sgRNA #3, the expected size of the PCR product was also 547 bp and cleavage by Cas9 resulted in fragments of 385 and 162 bp.

### 2.8 3D modelling of the KSR1 kinase domain

Protein binary interactions, functional sites, and experimental mutation data of eukaryotic protein kinases (EPKs) were retrieved from UniProt (www.uniprot.org). ModBase (http://modbase.compbio.ucsf.edu/), a database of comparative protein structure models, currently contains twenty‐two 3D‐models for mKSR1 covering the kinase domain (amino acids 563–833). Twenty 3D‐models are unreliable because of the sequence identity between mKSR1 and the proteins whose crystal structures were used as template, ranges from 12% to 28%, well below the threshold of 30% for reliable fold assignment. Therefore, we focussed our analysis on a 3D‐model of the mKSR1 region from amino acids 550–830, which possesses the highest sequence identity (71%) with human KSR2 (hKSR2). The mKSR1 3D‐model (550–830) was calculated using the crystallographic structure of hKSR2 (PDB ID: 2y4i, chain B) as a template [22]. We used the PDBeFold method to compare the mKSR1 3D‐model and crystal structures of other EPKs (https://www.ebi.ac.uk/msd‐srv/ssm/) [31]. The mKSR1 3D‐model superposes perfectly with hKSR2 and mouse cAMP‐dependent protein kinase catalytic subunit alpha (mPKA; PDB ID: 1atp, chain E; overall RMSD = 1.9 Å, overall *Q*‐score = 0.37, and 214 aligned residues; Fig. S2A). mPKA is a well‐studied EPK and is often used as a prototype for understanding the biophysical and biochemical properties of the entire kinome [32]). Based on the structural alignment by PDBeFold, and the experimental mutation data of hKSR2 and mPKA, we designed the mKSR1 mutant F701A, which is positioned in the aspartic acid–phenylalanine–glycine (DFG) motif and is the equivalent of mPKA‐F185.

## 3 Results

### 3.1 KSR proteins induce RAS‐independent proliferation via MAPK pathway activation

**Fig. 1:**
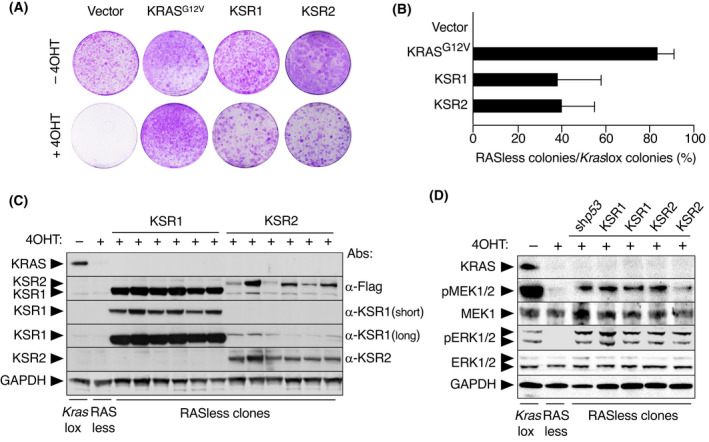
Ectopic expression of KSR1 or KSR2 sustains proliferation of RASless MEFs. (A) Representative images of a colony formation assay in *Kras*lox cells infected with retroviral vectors expressing the indicated cDNAs in the absence (–4OHT) or presence of 4OHT (+4OHT). (B) Quantification of colony formation in *Kras*lox and RASless MEFs expressing the indicated cDNAs expressed as percentage of colonies grown in RASless cells compared to those grown in *Kras*lox cells. Error bars indicate SD (*n* = 3 independent experiments). (C) Western blot analysis of Flag‐tagged KSR1, Flag‐tagged KSR2, and KRAS expression in *Kras*lox MEFs either left untreated (−), treated for 2 weeks with 4OHT (+) to generate RASless MEFs and RASless clones that grew after ectopic expression of KSR1 or KSR2. The expression of KSR proteins was detected using α‐Flag, α‐KSR1 (long and short exposure) or α‐KSR2 antibodies. The presence of 4OHT in the cultures to eliminate the expression of the endogenous KRAS protein is indicated. GAPDH expression served as loading control. The experiment has been repeated at least three times and one representative experiment is shown. (D) Western blot analysis of KRAS, p‐MEK1/2, MEK1, pERK1/2 and ERK1/2 expression in *Kras*lox MEFs, RASless MEFs, or representative RASless clones generated after ectopic expression of sh*p53*‐A (sh*p53*), KSR1 or KSR2. The presence of 4OHT in the cultures to eliminate expression of the endogenous KRAS protein is indicated. GAPDH expression served as loading control. The experiment has been repeated at least three times and one representative experiment is shown.

We have previously shown that activation of the MAPK pathway is sufficient to drive cell proliferation in the absence of RAS proteins [26]. To ascertain whether the KSR proteins may also induce cell proliferation in a RAS‐independent manner, we ectopically expressed KSR1 or KSR2 in *Hras* ^–/–^;*Nras* ^–/–^;*Kras* ^lox/lox^;RERT^ert/ert^ mouse embryonic fibroblasts (MEFs) either in the absence (*Kras*lox cells) or in the presence of 4OHT to ablate KRAS expression (RASless cells) [26]. As illustrated in Fig. 1A,B, the ectopic expression of KSR1 or KSR2 efficiently restored proliferation of RASless cells as determined by colony formation in the absence of RAS proteins (Fig. 1C). Analysis of KSR‐expressing RASless clones revealed consistently high levels of KSR1 and more variable levels of KSR2 expression, suggesting that clonal outgrowth in the absence of RAS proteins is compatible with a range of KSR expression levels, especially in the case of KSR2 (Fig. 1C). As expected, proliferating RASless clones due to the ectopic expression of KSR1 or KSR2 displayed an active MAPK pathway, as demonstrated by the phosphorylation of MEK and ERK (Fig. 1D). Considering that KSR1 and KSR2 promoted similar levels of cell proliferation when ectopically expressed in RASless cells, we focussed our analysis henceforth on KSR1, since this paralog had been studied in more detail.

### 3.2 Structural requirements for induction of RAS‐independent proliferation by KSR1

**Fig. 2:**
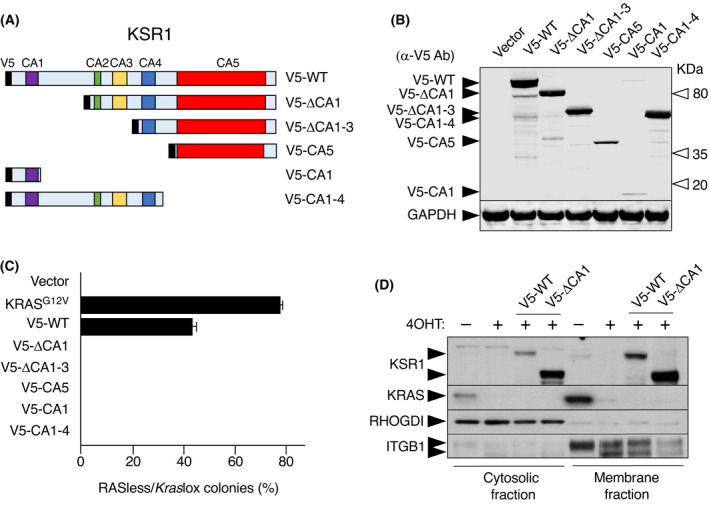
Full‐length KSR1 is required to induce proliferation of Rasless MEFs. (A) Schematic representation of wild‐type KSR1 or the indicated deletion mutants. Wild‐type KSR1 harbours 5 CAs: CA1 (including a coiled coil and sterile‐α‐motif; violet), CA2 (proline‐rich region; green), CA3 (cysteine‐rich motif; yellow), CA4 (serine/threonine‐rich domain; blue) and CA5 (kinase domain; red). The V5 tag is indicated as a black box. (B) Western blot analysis of wild‐type KSR1 or the indicated KSR1 deletion mutants fused to a V5‐tag probed with a V5 antibody. Migration of each of the indicated KSR1 mutants is indicated by black arrowheads. Open arrowheads indicate molecular weights. GAPDH expression served as a loading control. The experiment has been repeated at least three times and one representative experiment is shown. (C) Quantification of colony formation in *Kras*lox and RASless MEFs expressing the indicated cDNAs expressed as percentage of colonies grown in RASless cells compared to those grown in *Kras*lox cells. Error bars indicate SD (*n* = 3 independent experiments). (D) Western blot analysis of KSR1 and KRAS expression levels in *Kras*lox cells, RASless cells and RASless cells expressing wildtype KSR1 (V5‐WT) or KSR1^ΔCA1^ (V5‐ΔCA1) in the cytosolic and plasma membrane fractions. RHOGDI served as a marker for the cytosolic fraction and Integrin B1 (ITGB1) for the membrane fraction. The experiment has been repeated at least three times and one representative experiment is shown.

Next, we interrogated whether the activity of KSR1 was mediated by any of its conserved areas (CA1–5; Fig. 2A). CA1, located next to the N‐terminus of the protein, includes a region required for BRAF and MEK binding as well as a coiled coil and sterile‐α‐motif (CC‐SAM) proposed to be involved in association with membrane ruffles induced by EGF treatment [17, 33]. The other CAs include a proline‐rich domain (CA2), a cysteine‐rich motif also linked to membrane association (CA3), a serine/threonine‐rich region (CA4) and a putative kinase domain (CA5) highly homologous to corresponding kinase domains present in the RAF family of proteins [2, 16, 17, 25]. A series of KSR1 deletion mutants lacking the CA1, CA2/CA3 and CA4 domains fused to a V5 tag were generated (Fig. 2A,B). We also included constructs consisting only of the CA1 or the CA1–4. As illustrated in Fig. 2C, only the full‐length KSR1 protein was able to sustain proliferation of RASless MEFs. Surprisingly, we could not detect major differences in membrane association between full‐length KSR1 and the KSR1^ΔCA1^ deletion mutant, indicating that this domain is not essential for the interaction of KSR1 with the plasma membrane (Fig. 2D; Fig. S1A). Likewise, KSR1^ΔCA1^ still co‐precipitated with BRAF in *Kras*lox as well as in RASless cells, indicating that membrane attachment or association with BRAF is not sufficient for KSR to induce RAS‐independent proliferation (Fig. S1B).

### 3.3 KSR1 activity requires an intact DFG motif necessary for ATP binding

**Fig. 3:**
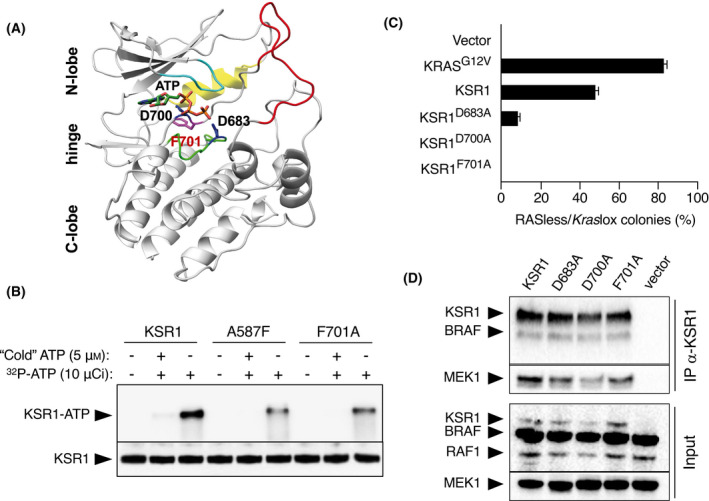
KSR1 requires ATP binding to induce proliferation of RASless MEFs. (A) 3D model of the KSR1 kinase domain in association with ATP in the active site. Residues D683, D700 and F701 are highlighted. The catalytic loop includes the HRD motif (green), the activation loop as an extension of the DFG motif (red), the G‐rich loop (light blue) and the αC helix (yellow). (B) ^32^P‐ATP binding to recombinant KSR1 or KSR1^F701A^. The KSR1^A587F^ mutant protein known to be defective in ATP binding was included as a control. The experiment has been repeated at least three times and one representative experiment is shown. (C) Quantification of colony formation in *Kras*lox and RASless MEFs expressing the indicated cDNAs as percentage of colonies grown in RASless cells compared to those grown in *Kras*lox cells. Error bars indicate SD (*n* = 3 independent experiments). (D) Immunoprecipitation using α‐KSR1 antibodies in 293T cells transfected with an empty vector or vectors expressing KSR1, KSR1^D683A^, KSR1^D700A^ or KSR1^F701A^. Immunoprecipitations (IP α‐KSR1) and total lysates (input) were probed with antibodies against KSR1, BRAF, RAF1 and MEK1. The experiment has been repeated at least three times and one representative experiment is shown.

Previous studies have indicated that KSR1 can bind ATP and displays kinase activity in spite of lacking the lysine residue required for the phosphotransfer reaction of most kinases [18, 20, 22]. Thus, we re‐examined whether we could detect these activities and if so, whether they play a role in RAS‐independent MAPK pathway activation. In a structural model of the KSR1 kinase domain, we noted that residue F701, which forms part of the conserved DFG motif, is likely to be involved in ATP binding (Fig. 3A; Fig. S2A–C). To evaluate the role of residue F701 in ATP binding and kinase activity, we affinity purified recombinant KSR1 and KSR1^F701A^ proteins expressed in mammalian cells (Fig. S3A–C). As a control, we also included the KSR1^A587F^ mutant protein known to be defective in ATP binding [21]. As predicted, KSR1^F701A^ had reduced capacity to bind ATP similarly to KSR1^A587F^, thus confirming that the F701 residue is required for efficient ATP binding (Fig. 3B). However, we were unable to detect kinase activity associated with purified KSR1 or KSR1^F701A^ mutant in *in vitro* assays (Fig. S3D). The expression of the mutant KSR^F701A^ isoform in RASless cells failed to induce proliferation, thus indicating that efficient ATP binding is essential for KSR1 to induce RAS‐independent proliferation (Fig. 3C).

We also interrogated the role of two additional residues thought to be essential for kinase activity, D683, which is part of the conserved histidine–arginine–aspartic acid (HRD) sequence within the catalytic loop of all kinases and D700, the residue adjacent to F701 within the conserved DFG sequence present in the kinase activation loop [19, 20, 34]. Whereas the KSR1^D683A^ isoform retained some residual proliferative activity, the D700A mutation completely abolished the capacity of KSR1 to induce RAS‐independent cell proliferation (Fig. 3C). Yet, KSR1^D683A^ and KSR1^D700A^ were expressed at slightly lower levels than KSR1^F701A^, which could, at least to some extent, affect their ability to induce RAS‐independent proliferation (Fig. 3D; Fig. S3E). In any case, the three mutant isoforms retained their ability to bind to BRAF and MEK1 (Fig. 3D).

### 3.4 KSR1, unlike RAF proteins, interacts with the plasma membrane in the absence of RAS proteins

**Fig. 4:**
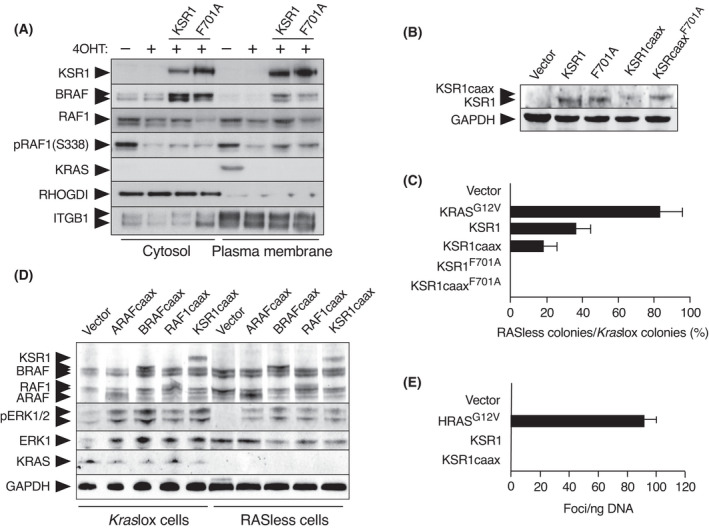
Membrane localization of KSR1 is not sufficient to induce RAS‐independent proliferation. (A) Western blot analysis of KSR1, BRAF, RAF1, pRAF1 (S338) and KRAS expression in *Kras*lox MEFs expressing cDNA encoding either wild type KSR1 or the mutant KSR1^F701A^ isoform (F701A) either left untreated (−), or treated for 2 weeks with 4OHT (+) to generate RASless MEFs. The cytosolic as well as the plasma membrane fraction is shown. Integrin B1 (ITGB1) served as a marker for the membrane fraction and RHOGDI for the cytosolic fraction. The experiment has been repeated at least three times, and one representative experiment is shown. (B) Western blot analysis of KSR1 expression in *Kras*lox cells expressing the indicated cDNAs. GAPDH expression served as a loading control. The experiment has been repeated at least three times and one representative experiment is shown. (C) Quantification of colony formation in *Kras*lox and RASless MEFs expressing the indicated cDNAs expressed as percentage of colonies grown in RASless cells compared to those grown in *Kras*lox cells. Error bars indicate SD (*n* = 3 independent experiments). (D) Western blot analysis of KSR1, ARAF, BRAF, CRAF, pERK1/2, ERK1 and KRAS expression in *Kras*lox MEFs expressing the indicated cDNAs either left untreated (−4OHT) or treated for 2 weeks with 4OHT (+4OHT) to generate RASless MEFs. The experiment has been repeated at least three times and one representative experiment is shown. (E) Quantification of focus formation in NIH3T3 cells transfected with plasmids expressing the indicated cDNA. Error bars indicate SD (*n* = 3 independent experiments).

We have previously reported that RAF proteins fail to induce proliferation of RASless cells unless they associate with the plasma membrane via insertion of the polybasic stretch and CAAX domain of KRAS proteins at their carboxy‐terminus [26]. Instead, KSR1 locates to the membrane fraction in RASless cells without the need to add a CAAX motif to the protein (Fig. 4A), an unexpected result considering that membrane localization of KSR was thought to depend on RAS activity [16, 17]. Interestingly, insertion of the inactivating F701A mutation into KSR1 did not affect its ability to associate with the plasma membrane, again reinforcing the concept that membrane association is not sufficient for KSR1 to drive cell proliferation (Fig. 4A). The expression of KSR1, and to a lesser extend also KSR1^F701A^, allows BRAF to associate with the plasma membrane fraction in RASless cells (Fig. 4A). RAF1 was also enriched in the plasma membrane fraction upon the expression of KSR1. Moreover, the expression of KSR1 was more efficient to activate RAF1 than KSR1^F701A^, as determined by the phosphorylation of Ser338 (Fig. 4A).

Interestingly, the addition of a CAAX motif to KSR1 did not improve its ability to sustain cell proliferation in the absence of RAS proteins (Fig. 4B,C). Instead, the KSR1caax isoform was less efficient, suggesting that the CAAX motif could “force” KSR1 to interact with the plasma membrane in a non‐optimal fashion. Moreover, unlike BRAFcaax or RAF1caax proteins, KSR1caax failed to transform NIH3T3 cells despite inducing similar levels of ERK phosphorylation as RAF kinases fused to a CAAX box in RASless cells (Fig. 4D,E). The addition of the CAAX box motif to the KSR1^F701A^ mutant isoform also failed to induce cell proliferation (Fig. 4B,C). These observations indicate that efficient ATP binding along with its intrinsic ability to interact with the plasma membrane are essential requirements for KSR1 to sustain cell proliferation in the absence of RAS proteins.

### 3.5 KSR1 heterodimerization with RAF kinases is essential to sustain cell proliferation in the absence of RAS proteins

**Fig. 5:**
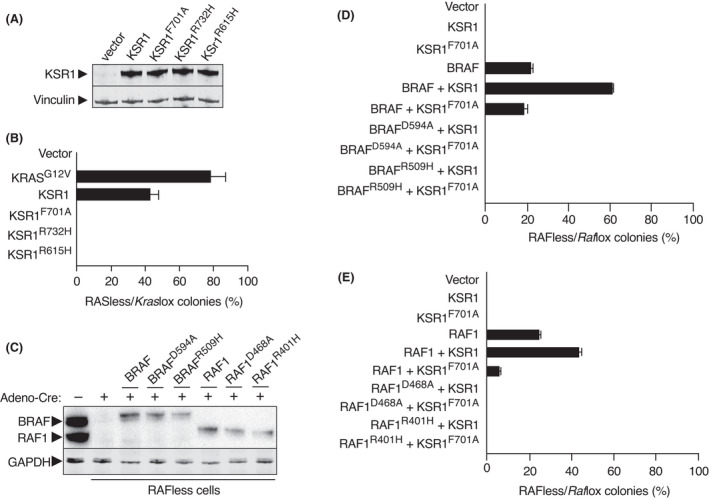
KSR induces proliferation in RASless MEFs by heterodimerization with RAF proteins. (A) Western blot analysis of KSR1 expression in *Kras*lox cells expressing cDNAs encoding the indicated KSR1 mutants. GAPDH expression served as a loading control. The experiment has been repeated at least three times and one representative experiment is shown. (B) Quantification of colony formation in *Kras*lox and RASless MEFs expressing cDNAs encoding KRAS^G12V^ as positive controls as well as the indicated KSR1 isoforms. Results are expressed as the percentage of colonies grown in RASless cells compared to those grown in *Kras*lox cells. Error bars indicate SD (*n* = 3 independent experiments). (C) Western blot analysis of BRAF and RAF1 expression in untreated *Araf* ^lox/lox^;*Braf* ^lox/lox^;*Raf1* ^lox/lox^ (*Raf*lox) MEFs (−) and in *Raf*less MEFs generated after infection with Adeno‐Cre and expressing cDNAs encoding the indicated BRAF and RAF1 isoforms. The experiment has been repeated at least three times and one representative experiment is shown. (D, E) Quantification of colony formation in *Raf*lox and RAFless MEFs expressing cDNAs encoding the indicated BRAF and RAF1 isoforms, respectively. Results are expressed as percentage of colonies grown in RAFless cells compared to those grown in *Raf*lox cells. Error bars indicate SD (*n* = 3 independent experiments).

To ascertain the mechanisms by which KSR1 directs RAF proteins to the plasma membrane, we introduced mutations within KSR1 (KSR1^R732H^ and KSR1^R615H^) known to perturb its interaction with BRAF and possibly also with RAF1 [23, 35]. As shown in Fig. 5A,B, the introduction of either mutation into KSR1 completely eliminated its colony‐forming capacity in RASless cells. Thus, this suggests that KSR1 must retain its capacity to dimerize with RAF kinases to induce RAS‐independent proliferation. Likewise, the ectopic expression of KSR1 failed to induce proliferation in cells lacking *Raf1, Araf* and *Braf* alleles (RAFless cells) [27] (Fig. 5C–E), indicating that KSR proteins cannot activate MEK kinases on their own, in spite of the fact that previous studies have indicated that KSR1 can phosphorylate MEK proteins [20]. These observations indicate that KSR1 requires the presence of RAF proteins and the capacity to heterodimerize with them to stimulate the activation of the MAPK pathway.

Next, we introduced wild‐type or ATP binding‐deficient KSR1^F701A^ together with wild‐type BRAF or RAF1 into RAFless cells and assayed for colony formation. As illustrated in Fig.  5C–E, wild‐type KSR1, but not KSR1^F701A^, enhanced the capacity of both BRAF and RAF1 to sustain the proliferation of RAFless cells. Again, this confirmed that ATP binding is required for KSR proteins to stimulate RAF activity. To ascertain whether the kinase activity of RAF was also required, we replaced wild‐type BRAF or RAF1 with kinase‐inactive BRAF^D594A^ and RAF1^D468A^ isoforms, respectively. None of the kinase‐inactive RAF proteins was able to sustain the proliferation of RAFless cells even in the presence of KSR1 (Fig. 5C–E).

Finally, we replaced wild‐type BRAF or RAF1 with the respective dimerization‐deficient BRAF^R509H^ and RAF1^R401H^ mutants known to be defective in dimerizing with KSR [23, 24]. As shown in Fig. 5C–E, dimerization‐defective RAF proteins also failed to induce the proliferation of RAFless cells. Together, these data suggest that KSR proteins, when expressed at significant levels, induce MAPK signalling and RAS‐independent proliferation via the activation of RAF kinases by a mechanism involving heterodimerization between these proteins. Yet, heterodimerization between KSR and RAF proteins was necessary but not sufficient, since it also required an intact DFG motif within KSR for efficient ATP binding. Whether a putative kinase activity of KSR1 is also implicated remains to be resolved.

### 3.6 KSR induced proliferation of RASless cells is independent of p53 inactivation

We have previously described that RASless cells can also proliferate upon loss of the p53 tumour suppressor by a mechanism dependent on the presence of RAF proteins [27]. To determine whether there was a direct mechanistic connection between KSR1 activity and loss of p53, we treated KSR1 expressing RASless cells with doxorubicin to induce DNA damage and, subsequently, p53 activation. As shown in Fig. S4A, KSR1‐expressing RASless cells retained functional p53, thus suggesting that KSR1 induces proliferation through a mechanism that does not involve p53 inactivation. Vice versa, we interrogated whether p53 ablation might result in increased levels of KSR1 expression similar to those observed upon ectopic expression of this protein. As illustrated in Fig. S4B, the absence of p53 expression had no effect on the low levels of expression of KSR1 in RASless cells. Moreover, the concomitant elimination of KSR1 and p53 did not alter RAS‐independent colony formation that resulted upon p53 elimination alone (Fig. S4C,D). Finally, KSR2 did not play any role in this process since this protein in not expressed in MEFs [36].

### 3.7 High KSR expression levels reduce the efficacy of KRAS^G12C^ inhibitors

**Fig. 6:**
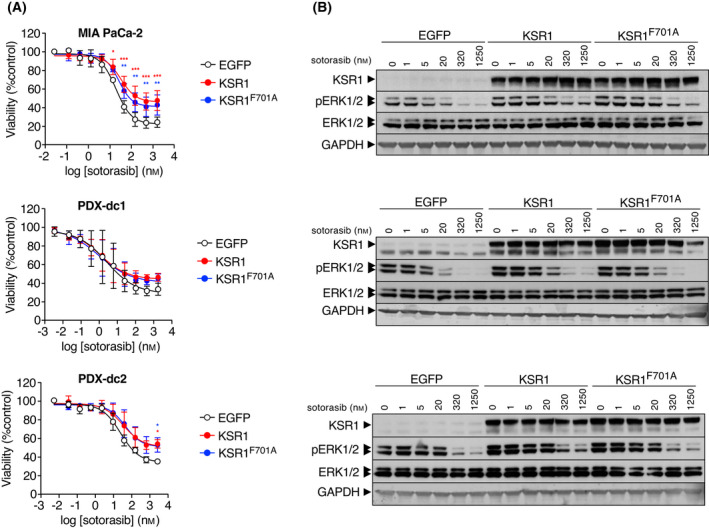
KSR1 expression decreases the responsiveness to sotorasib treatment. (A) Relative viability of MIA PaCa‐2, PDX‐dc1 and PDX‐dc2 cells expressing EGFP (open circles), KSR1 (red) or KSR1^F701A^ (blue) proteins after treatment with the indicated doses of sotorasib for 72 h. Error bars indicate SD (*n* = 3 independent experiments). *P < 0.05; **P < 0.001, ***P > 0.001 (two‐way ANOVA with Dunnett's multiple comparison test) (B) Western blot analysis of KSR1, pERK1/2 and ERK1/2 expression levels in MIA PaCa‐2, PDX‐dc1 and PDX‐dc2 cells expressing EGFP, KSR1 or KSR1^F701A^ proteins after treatment with the indicated doses of AMG 510 for 24 h. GAPDH expression served as a loading control. The experiment has been repeated at least three times and one representative experiment is shown.

Recently, two KRAS oncogene inhibitors have been either approved (sotorasib, AMG510) or given breakthrough therapy designation (adagrasib, MRTX849) by the FDA. Both of these inhibitors require the formation of a covalent bond with the cysteine residue responsible for the oncogenic properties of a KRAS^G12C^ mutant isoform [37, 38, 39]. Based on the ability of KSR proteins to induce cell proliferation in the absence of RAS proteins, we examined whether increased levels of KSR may modulate the responsiveness of KRAS^G12C^ mutant cells to these inhibitors. To this end, we ectopically expressed KSR1 or KSR1^F701A^ proteins in human tumour cells known to express the KRAS^G12C^ oncoprotein including MIA PaCa‐2 cells as well as two cell lines derived from PDX established from lung tumours, PDX‐dc1 and PDX‐dc1 [29]. As shown in Fig. 6A, the ectopic expression of KSR1 caused sotorasib to inhibit proliferation in all three cell lines with significantly lower efficacy. Surprisingly, the expression of KSR1^F701A^ caused a similar effect as wild‐type KSR1. Ectopic KSR1 expression also caused higher basal levels of ERK phosphorylation as well as increased pERK levels at higher sotorasib concentrations (Fig. 6B). Furthermore, KSR1 expression levels correlated with increasing concentrations of the drug in sotorasib‐resistant MIA PaCa‐2 as well as PDX‐dc1 cell lines (Fig. S5A,B). Taken together, these data suggest that elevated levels of KSR1 in human tumours may reduce the efficacy of KRAS inhibitors due to the ability of KSR proteins to activate the MAPK in a RAS‐independent manner.

## 4 Discussion

Kinase suppressor of RAS proteins have long been considered simply scaffold proteins required for optimal MAPK signalling output [1, 2]. Yet, increasing evidence suggests that KSR proteins exert additional activities independently of their scaffolding role [18]. Here, we demonstrate that KSR proteins play a more complex role in MAPK signalling than originally anticipated. Indeed, the ectopic expression of KSR1 or KSR2 is sufficient to license the proliferation of RASless cells via RAS‐independent activation of the MAPK pathway. Our results indicate that the mechanism by which KSR proteins activate the MAPK pathway in the absence of RAS proteins involves their heterodimerization with RAF proteins, an event that presumably facilitates the recruitment of the latter to the plasma membrane where they become activated. This activation step also requires efficient ATP binding by KSR.

Previous studies from our laboratory have demonstrated that MEFs can proliferate normally in the absence of RAS proteins as long as the p53/p21^Cip1^/Rb tumour suppressor pathway is inactivated [27]. Indeed, RAS proteins also contribute to cellular proliferation by a MAPK independent mechanism that involves the inactivation of p53‐induced p21^Cip1^ expression via the inhibition of acetylation of critical p53 lysine residues [27]. Yet, cellular proliferation in the absence of the p53/p21/Rb axis requires the presence of RAF proteins. Here, we demonstrate that the proliferative role of KSR proteins in the absence of RAS does not involve inactivation of p53. Likewise, loss of p53 does not result in increased levels of expression of KSR proteins. These observations illustrate that there are at least two independent mechanisms by which cells can proliferate in the absence of RAS proteins, loss of the p53/p21^Cip1^/Rb axis and increased KSR expression. Yet, in both cases, the presence of RAF proteins as well as their translocation to the plasma membrane are required [26, 27, 30], thus, indicating that RAF proteins are the key effectors of the MAPK pathway. Whether other regulatory events could induce the proliferation of MEFs in the absence of RAF proteins remains to be determined.

It has been proposed that, at least *in vitro*, KSR1 can phosphorylate MEK1 in its activation segment in a manner similar to RAF proteins [20]. Yet, we could not obtain biochemical evidence for such an activity. Moreover, the genetic ablation of the three RAF proteins prevented cellular proliferation upon ectopic KSR expression. Since their discovery, it has been controversial whether KSR proteins are active kinases, since both KSR1 and KSR2 lack, among other amino acids, a highly conserved lysine residue in their kinase domain [16, 17, 18]. However, the crystal structure of the KSR2 kinase domain with MEK1 revealed the presence of ATP within the ATP‐binding pocket [22]. Moreover, the same study demonstrated in *in vitro* assays, the phosphorylation of MEK1 mediated by KSR2 at residues in the N‐terminal part of the protein that are believed to facilitate MEK phosphorylation by RAF kinases. Likewise, ATP binding was also required for efficient ERK activation and RAS‐mediated transformation [21].

Our data indicate that mutations within the DFG motif of KSR that perturb ATP binding abolish proliferation, suggesting that efficient allosteric RAF activation may require ATP binding. It has been proposed that allosteric RAF activation by KSR requires formation of a side‐to‐side dimer of their respective kinase domains [23], arguing that such dimer may not properly form when ATP binding to KSR is reduced. It is possible that a functional DFG motif as well as ATP binding are necessary to induce assembly of the regulatory hydrophobic (R−) spine on RAF [40]. Indeed, Hu and colleagues demonstrated that ATP binding‐deficient and presumably kinase‐inactive KSR1^A587F^ can dimerize with RAF but not activate it [40]. Yet, mutations in or close to the N‐terminal acidic (NtA) motif of KSR1 that mimic phosphorylation of selective amino acids render ATP binding‐deficient KSR1^A587F^ capable of activating RAF [40]. These observations suggest that when KSR levels are high, its NtA may become phosphorylated to act as an activator of RAF. This also means that the putative NtA phosphorylation of KSR could be a highly regulated process that can be bypassed when KSR expression levels rise above a certain threshold.

Our data do not explain the precise mechanisms by which KSR1 translocates to the membrane in the absence of RAS. It has been proposed that binding of MEK1 to KSR opens the closed, auto‐inhibited conformation of KSR1 and stimulates the binding and allosteric activation of BRAF via its CC‐SAM domain [24]. In this model, however, RAS activation and binding to BRAF is thought to be a prerequisite to open BRAF's auto‐inhibited conformation to license proliferation [41]. Therefore, our data indicate that ATP binding‐competent KSR molecules should somehow be able to open the closed inactive conformation of RAF kinases in the absence of RAS proteins, an event that could be mediated by the formation of heterodimers between RAF and KSR proteins. They also suggest that although the CC‐SAM domain located within CA1 seems not to be essential for BRAF binding, it may well be required for allosteric BRAF activation via assembly of the R‐spine. Finally, other domains such as the CA3 have also been implicated in membrane association [25, 42]. Which domain is ultimately required for membrane re‐localization in RASless cells remains to be determined.

Several crucial steps during RAF kinase activation occur at the membrane [41]. In fact, fusing the CAAX motif of KRAS to any of the RAF kinases is sufficient for their constitutive activation and induction of RAS‐independent proliferation [26]. These results indicate that opening of the inactive conformation of RAF kinases can occur at the membrane by a RAS‐independent mechanism, at least in MEFs. Since, as described above, KSR can directly associate with membranes through several structural motifs [33, 42], it is likely that KSR proteins recruit RAF kinases to the plasma membrane, where they are converted into their active state. KSR is usually sequestered in the cytoplasm where binding to 14‐3‐3 and IMP prevents its membrane localization [13]. Upon RAS activation, 14‐3‐3 and IMP displace from KSR, allowing it to translocate to the membrane. Hence, our data propose that ectopic KSR expression levels may out‐titrate the inhibitory activity of 14‐3‐3 and IMP allowing its constitutive membrane localization and subsequent RAF activation (for a model see Fig. S6).

High expression levels of KSR may also render KRAS inhibitors less effective. Since several inhibitors targeting KRAS oncoproteins carrying the G12C mutation have reached the clinic [37, 38, 39], our model (Fig. S6) raises a note of caution that high KSR expression levels may decrease tumour sensitivity to these as well as to forthcoming KRAS inhibitors. Indeed, our data show that high KSR1 expression levels reduces the efficacy of the KRAS^G12C^ inhibitor sotorasib in human cancer cells. While efficient ATP binding of KSR1 is required for the induction of RAS‐independent proliferation in MEFs, it seems to be less relevant for the reduced response to KRAS inhibitors in cancer. Although the reason for this discrepancy is currently unclear, the induction of RAS‐independent proliferation in MEFs may be a more stringent event that requires optimal KSR1 activity. Notably, KSR2 has also been linked to MAPK pathway reactivation and resistance to BRAF+MEK inhibition in melanoma cells, thus suggesting that the roles of KSR1 or KSR2 in modulating the response to KRAS/MAPK pathway inhibition may have been underestimated [43]. Indeed, an inhibitor stabilizing the inactive conformation of KSR has recently been described that enhanced the effects of MEK inhibition, although it remains to be determined whether this inhibitor synergizes with KRAS inhibitors [44]. Additional research is required to better understand the roles of KSR proteins in cancer.

## 5 Conclusions

Activated RAS proteins play a critical role in cancer and consequently tremendous efforts are being dedicated to devise therapeutic strategies to inhibit the oncogenic signals mediated by these oncoproteins. This goal requires a profound understanding of the molecular events that result for RAS oncogenic signalling. A potential strategy to understand the role of those downstream targets that mediate RAS signalling is to systematically study their properties in the absence of RAS proteins. Accumulating evidence indicates that the activation of the MAPK pathway is both necessary and sufficient to promote cell proliferation downstream of RAS. Indeed, direct MAPK pathway activation at any level can induce RAS‐independent proliferation and hence resistance to RAS inhibition. The regulation of MAPK pathway activity is complex, and numerous factors can modulate its activity. KSR1 and KSR2 were initially discovered as pseudokinases acting as scaffolding proteins for the MAPK pathway, but subsequent studies have proposed that they can play more active roles in signal transduction. Our data presented in this manuscript show that KSR1 and KSR2, when expressed at high levels, can directly activate the MAPK pathway and promote RAS‐independent proliferation by heterodimerizing with RAF proteins and allosterically stimulating their activation. These results suggest that high KSR1 or KSR2 expression levels in tumours could render strategies aimed at inhibiting RAS largely ineffective. Indeed, we further show that KRAS^G12C^ inhibitors are less effective when KSR1 expression levels are elevated. In conclusion, our data should raise awareness that the effectiveness of RAS inhibition is directly modulated by KSR1 or KSR2 expression levels.

## Conflict of interest

The authors declare no conflict of interest.

## Author contributions

GP, HKCJ, OB, SGA, CGL, TP and MD performed experiments and analysed the data. MM and CG provided critical reagents. MD and MB conceptualized the study, supervised the work and wrote the paper. MB acquired funding.

### Peer review

The peer review history for this article is available at https://publons.com/publon/10.1002/1878‐0261.13213.

## Supporting information


**Fig. S1**. KSR1^ΔCA1^ localizes to the plasma membrane and binds BRAF.
**Fig. S2**. 3D model of the mKSR1 kinase domain.
**Fig. S3**. Purification of recombinant KSR1 protein.
**Fig. S4**. RAS‐independent proliferation in the absence of p53 does not involve KSR.
**Fig. S5**. Western blot analysis of KSR1 expression levels in parental and resistant MIA PaCa‐2 (A) as well as PDX‐dc1 (B) cell lines.
**Fig. S6**. Model of KSR‐driven proliferation in RASless cells.

## Data Availability

All data are presented within the main figures and the supporting information of this manuscript.
