# Supplementary material for: KSR induces RAS‐independent MAPK pathway activation and modulates the efficacy of KRAS inhibitors
Source: Mol Oncol. 2022 Apr 12;16(17):3066–81. doi: 10.1002/1878-0261.13213 (PMC9441002; doi:10.1002/1878-0261.13213)
Supplement: Supplementary file 1 — Fig. S1. KSR1ΔCA1 localizes to the plasma membrane and binds BRAF. Fig. S2. 3D model of the mKSR1 kinase domain. Fig. S3. Purification of recombinant KSR1 protein. Fig. S4. RAS‐independent proliferation in the absence of p53 does not involve KSR. Fig. S5. Western blot analysis of KSR1 expression levels in parental and resistant MIA PaCa‐2 (A) as well as PDX‐dc1 (B) cell lines. Fig. S6. Model of KSR‐driven proliferation in RASless cells. [file MOL2-16-3066-s001.pdf]

Fig. S1

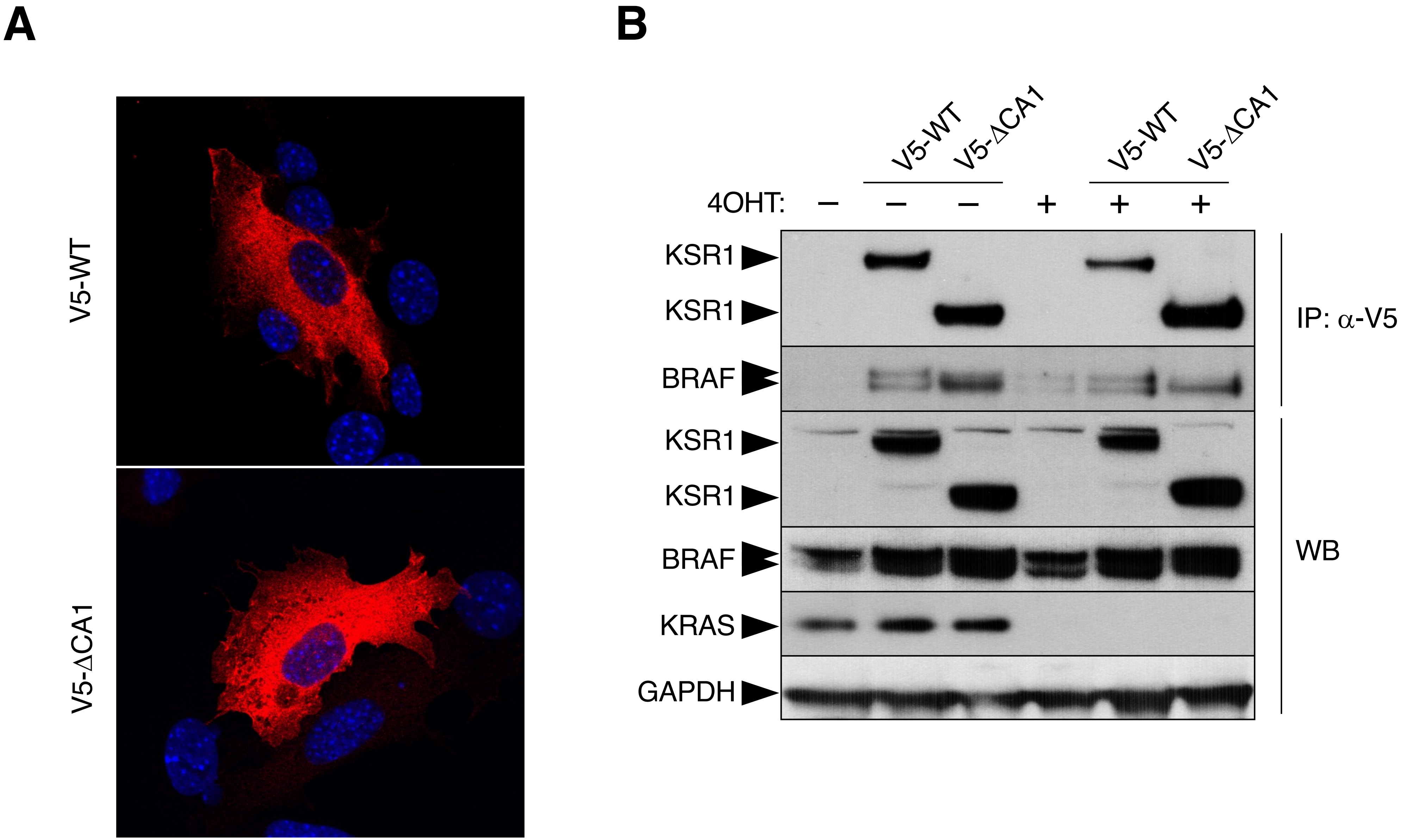
A
V5-WT
V5-ΔCA1
B
V5-WT
V5-ΔCA1
V5-WT
V5-ΔCA1
4OHT: – – – + + +
KSR1
KSR1
BRAF
KSR1
KSR1
BRAF
KRAS
GAPDH
IP: α-V5
WB

Figure S1. KSR1$^{\Delta CA1}$ localizes to the plasma membrane and binds BRAF. (A) Representative immunofluorescence staining of wild-type KSR1 (V5-WT) or KSR1$^{\Delta CA1}$ (V5-ΔCA1) in RASless cells using α-V5 antibodies (red). Hoechst 33352 counterstaining was used to highlight nuclei. (B) Western blot analysis of KSR1 and BRAF expression in untreated *Kras*lox cells (-) expressing wild-type KSR1 (V5-WT) or KSR1$^{\Delta CA1}$ (V5-ΔCA1) as well as in RASless cells (+) expressing the same cDNAs after immunoprecipitation with α-V5 antibodies (IP: α-V5). Expression of KSR1, BRAF and KRAS in total protein lysates is also shown (WB). GAPDH expression served as a loading control for total lysates.

Fig. S2

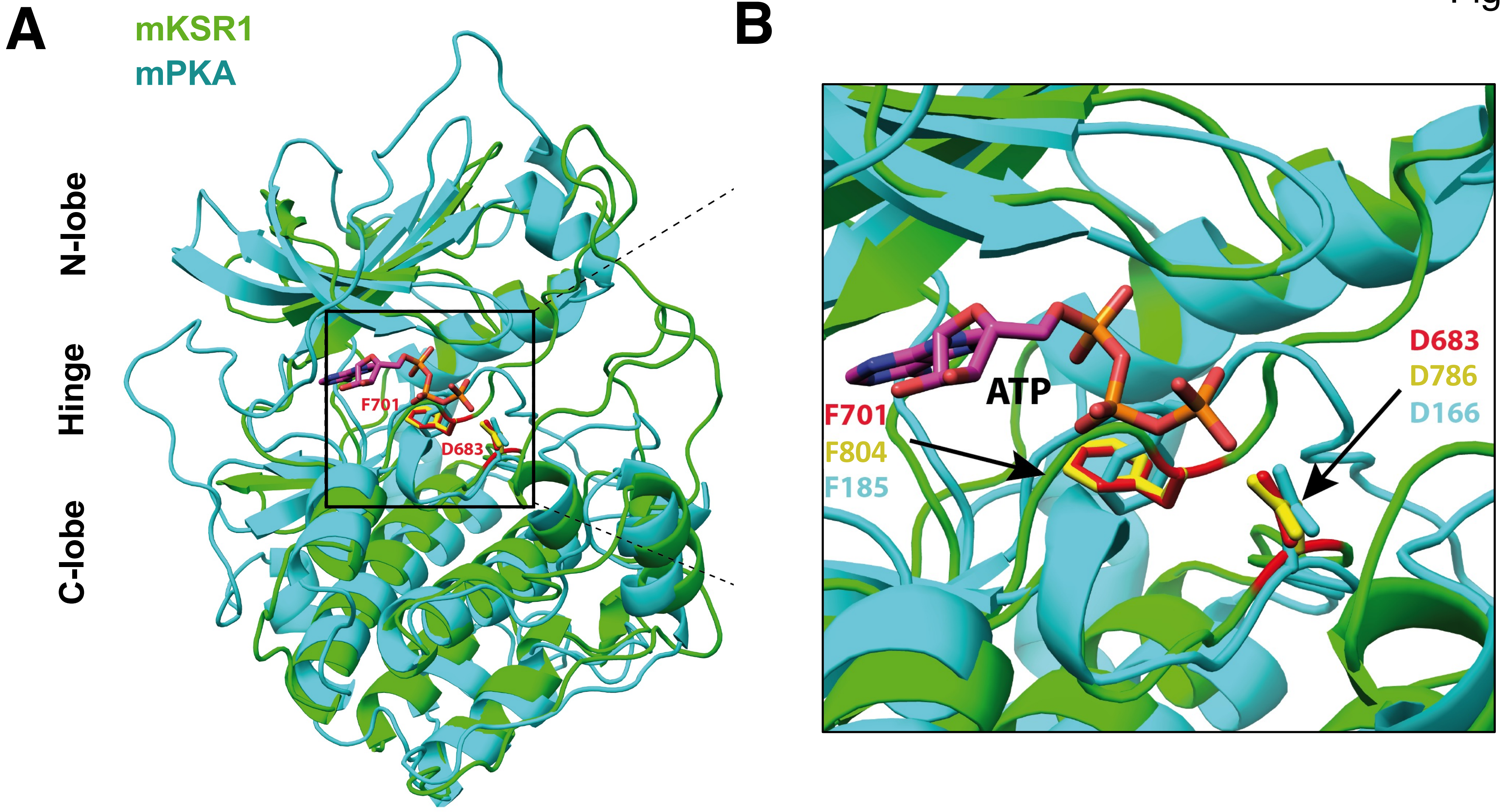

C

3D ALIGNMENT OF BACKBONE ATOMS

PDBeFold v2.59
RMSD = 1.8 Angstroms
Alignment length = 214 residues

***H/YRD motif** (**catalytic loop**)*

mKSR1		hKSR2		mPKA
HIS 681	|*|	HIS 784	|*|	TYR 164
LYS 682	|*|	LYS 785	|*|	ARG 165
**ASP 683**	|*|	**ASP 786**	|*|	ASP 166

DFG motif

mKSR1		hKSR2		mPKA
ASP 700	|*|	ASP 803	|*|	ASP 184
**PHE 701**	|*|	PHE 804	|*|	**PHE 185**
gly 702	| |	gly 805	| |	
LEU 703	|*|	LEU 806	|*|	GLY 186

Figure S2. 3D model of the mKSR1 kinase domain. (A) Left: Structural superposition of mKSR1 (green), hKSR2 (yellow), and mPKA (light blue). Since hKSR2 was used to model the structure of mKSR1, both structures perfectly overlap when the side chains are not shown. Phenylalanine (F) 701 and Aspartic acid (D) 683 in mKSR1 are indicated in red. ATP is also shown. Right: Magnification of the ATP-binding domain. F701 and D683 in mKSR1 are shown in red, F804 and D786 in hKSR2 are indicated in yellow and F185 as well as D166 in mPKA are shown in light blue. (B) PDBeFold results for the H/YRD and DFG motifs. Residues D683 and F701 in mKSR2 are shown in red, and structural equivalents D786 (hKSR2) and D166 (mPKA) as well as F804 (hKSR2) and F185 (mPKA) are highlighted.

Fig. S3

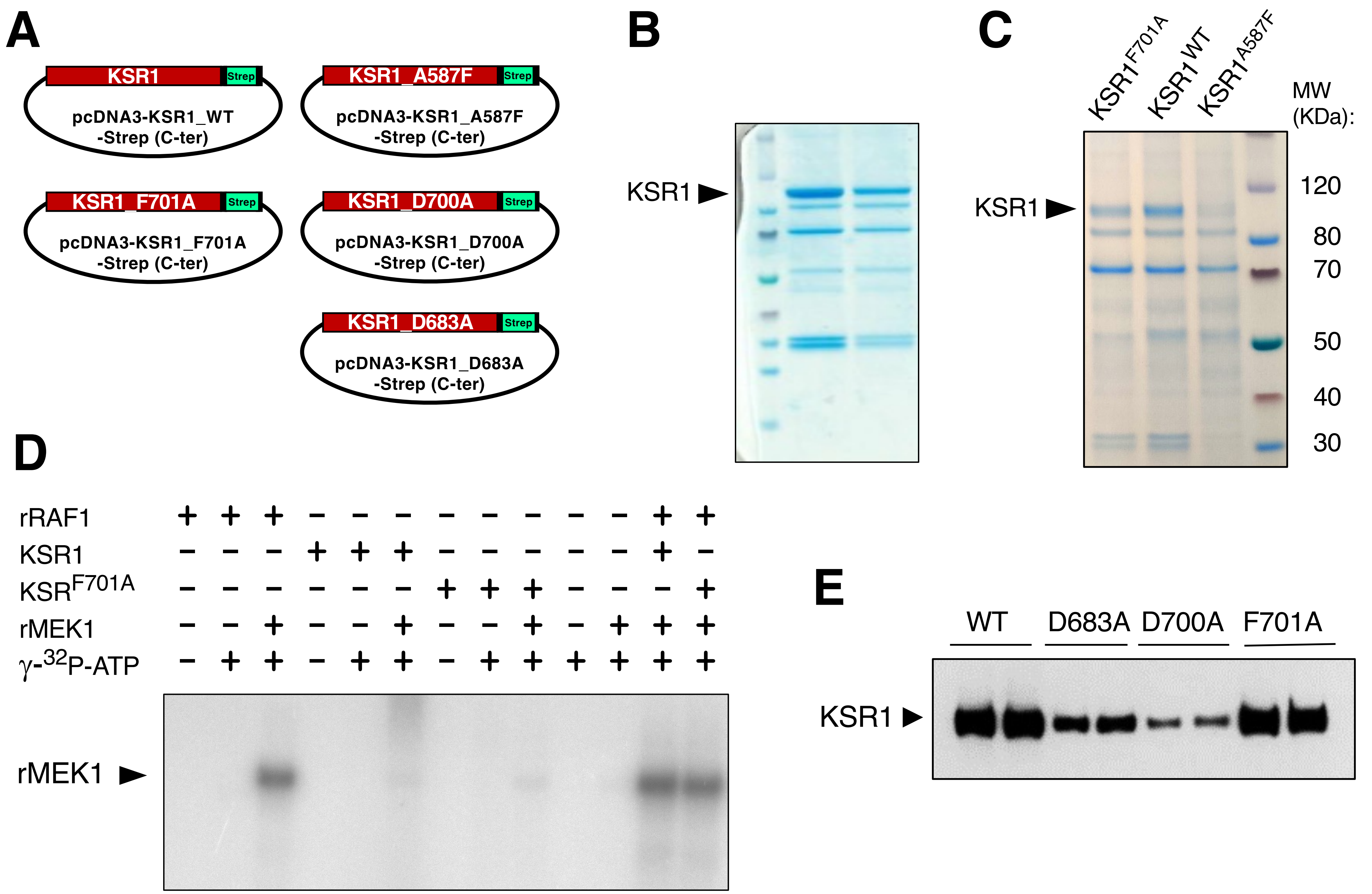

A
KSR1
Strep
pcDNA3-KSR1_WT
-Strep (C-ter)
KSR1_A587F
Strep
pcDNA3-KSR1_A587F
-Strep (C-ter)
KSR1_F701A
Strep
pcDNA3-KSR1_F701A
-Strep (C-ter)
KSR1_D700A
Strep
pcDNA3-KSR1_D700A
-Strep (C-ter)
KSR1_D683A
Strep
pcDNA3-KSR1_D683A
-Strep (C-ter)
B
KSR1
C
KSR1 F701A
KSR1 WT
KSR1 A587F
MW
(KDa):
120
80
70
50
40
30
KSR1
D
rRAF1 + + + − − − − − − − − + +
KSR1 − − − + + + − − − − − + −
KSR F701A − − − − − − + + + − − − +
rMEK1 − − + − − + − − + − + + +
γ-32P-ATP − + + − + + − + + + + + +
rMEK1
E
WT
D683A
D700A
F701A
KSR1

Figure S3. **Purification of recombinant KSR1 protein** (A) Schematic depiction of expression vectors used to express mouse KSR1 and the indicated mutant KSR isoforms fused to a C-terminal StrepTagII. (B) Coomassie-brilliant blue staining of purified mouse KSR1 and KSR^{F701A} proteins. (C) Coomassie-brilliant blue staining of purified mouse KSR1, KSR^{F701A} and KSR^{A587F} (D) Representative kinase assay using 0.1 μg recombinant RAF1 (rRAF1), 2 μg purified KSR1, 2 μg purified $KSR1^{F701A}$, 2 μg recombinant MEK1 (rMEK1) as a substrate and 10 μCi γ-^{32}P-ATP. (E) Western blot analysis of purified mouse KSR1, KSR^{D683A}, KSR^{D700A} and KSR^{F701A}. Expi293 cells were transfected with equal amounts of expression vectors and equal amounts of protein were loaded.

Fig. S4

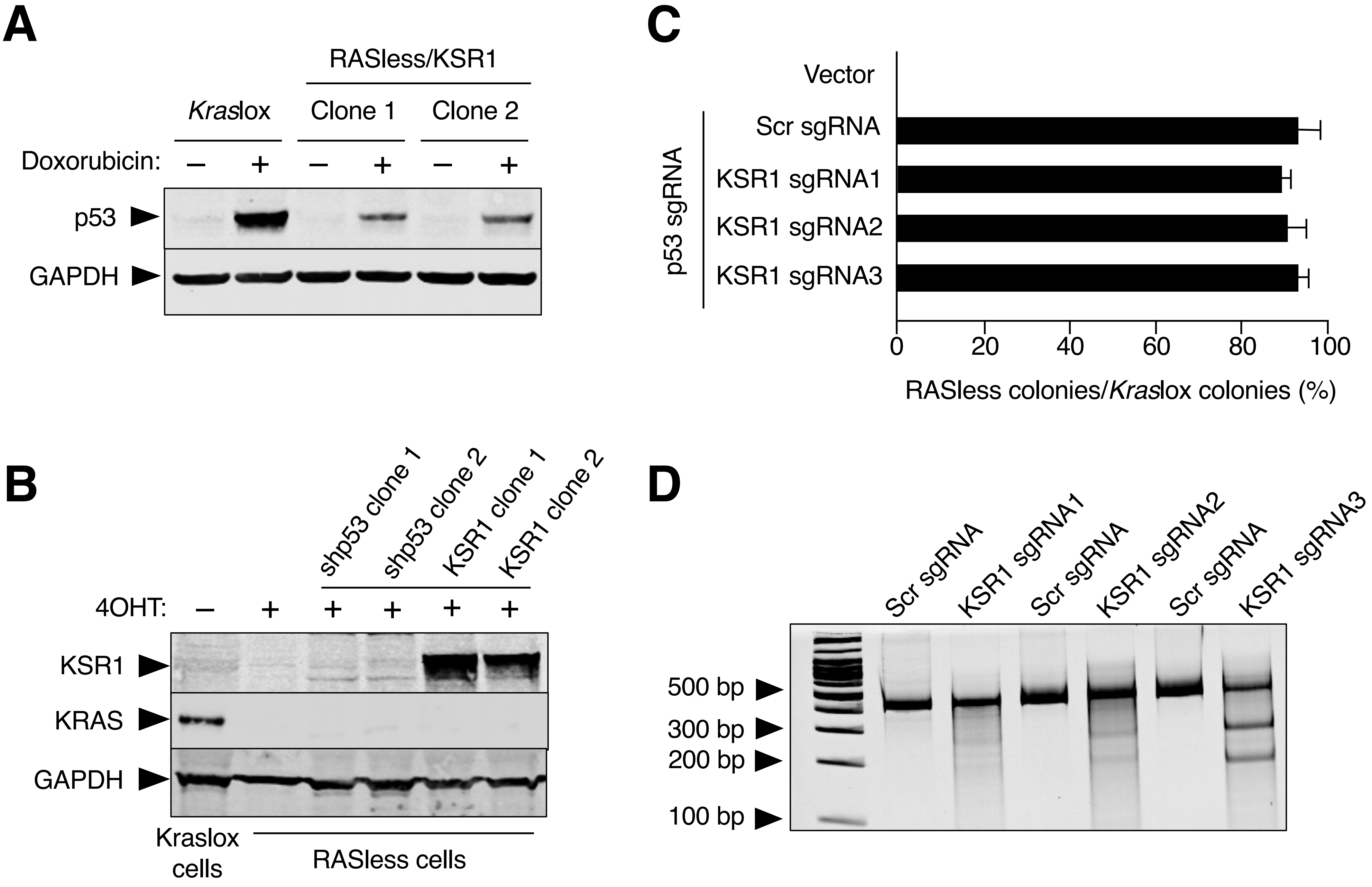
A
RASless/KSR1
Kraslox
Clone 1
Clone 2
Doxorubicin:
–
+
–
+
–
+
p53
GAPDH
B
shp53 clone 1
shp53 clone 2
KSR1 clone 1
KSR1 clone 2
4OHT:
–
+
+
+
+
+
KSR1
KRAS
GAPDH
Kraslox cells
RASless cells
C
Vector
p53 sgRNA
Scr sgRNA
KSR1 sgRNA1
KSR1 sgRNA2
KSR1 sgRNA3
0
20
40
60
80
100
RASless colonies/Kraslox colonies (%)
D
Scr sgRNA
KSR1 sgRNA1
Scr sgRNA
KSR1 sgRNA2
Scr sgRNA
KSR1 sgRNA3
500 bp
300 bp
200 bp
100 bp

Figure S4. RAS-independent proliferation in the absence of p53 does not involve KSR (A) Western blot analysis of p53 expression in *Kras*lox cells and two independent RASless clones that grew after ectopic expression of KSR1 either untreated (–), or after treatment with 5µg/ml doxorubicin for 16h (+). GAPDH expression served as loading control. (B) Western blot analysis of KSR1 and KRAS expression in *Kras*lox cells left untreated (–), treated for 2 weeks with 4OHT (+) to generate RASless MEFs and two independent RASless clones that grew after depletion of p53 or ectopic expression of KSR1. The presence of 4OHT in the cultures to eliminate expression of the endogenous KRAS protein is indicated. GAPDH expression served as loading control. (C) Quantification of colony formation in *Kras*lox and RASless MEFs expressing the indicated sgRNAs expressed as percentage of colonies grown in RASless cells compared to those grown in *Kras*lox cells. Viral vectors expressing the *p53* sgRNA and the Cas9 protein were selected with blasticidin and viral vector expressing scrambled (Scr) or *Ksr1* sgRNA were selected with puromycin. Error bars indicate standard deviation. (D) T7 endonuclease assay confirming Cas9-mediated Indel formation in the *Ksr1* locus in *Kras*lox cells after expression of scrambled (Scr) or 3 different *Ksr1* sgRNAs.

Fig. S5

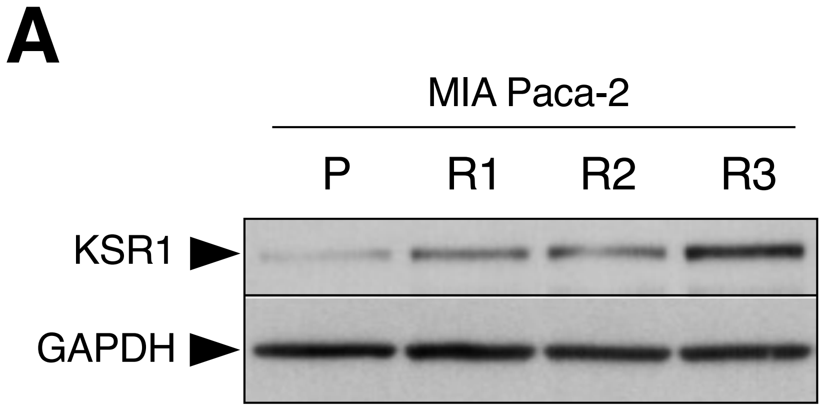

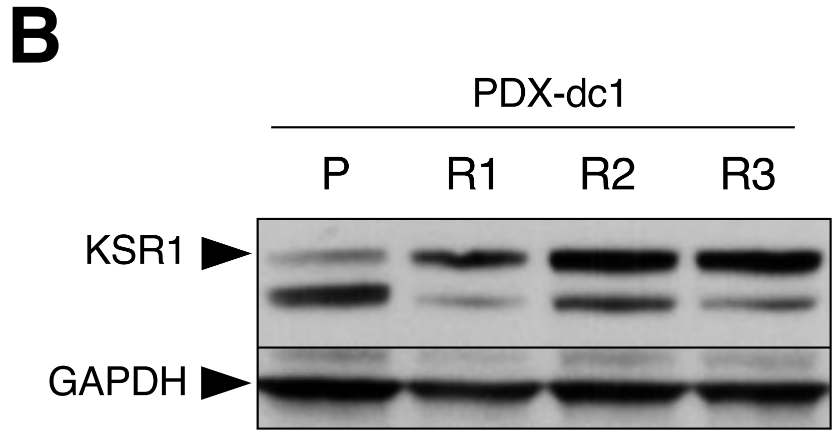

Figure S5. Western blot analysis of KSR1 expression levels in parental and resistant MIA PaCa-2 (A) as well as PDX-dc1 (B) cell lines. P, parental cell line; R1, resistant cells obtained after continuous exposure to 1 μM sotorasib; R2, resistant cells obtained after continuous exposure to 4 μM sotorasib; R3, resistant cells obtained after continuous exposure to 16 μM sotorasib. GAPDH expression served as a loading control.

Fig. S6

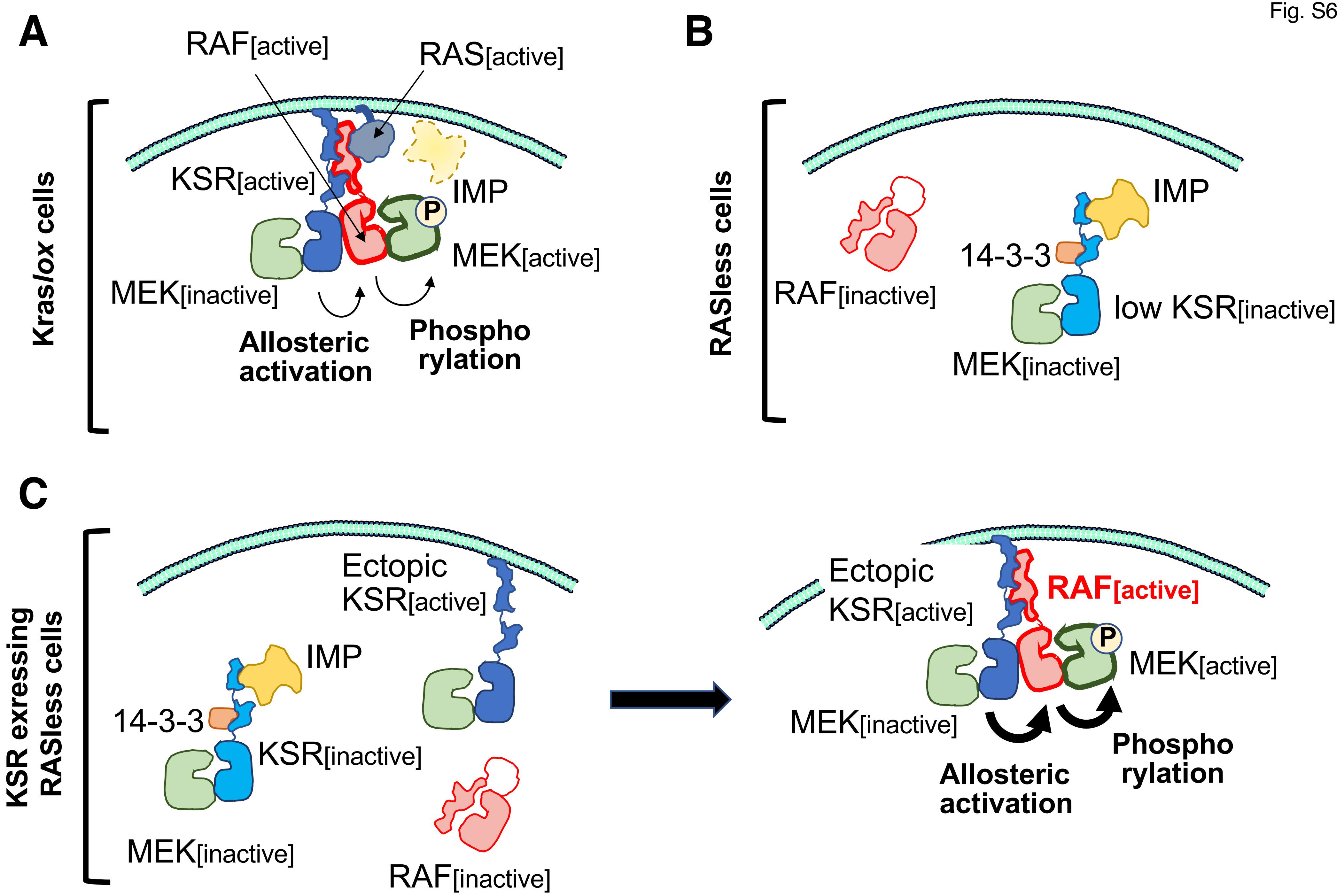
A
RAF[active]
RAS[active]
KSR[active]
IMP
P
MEK[active]
MEK[inactive]
Allosteric activation
Phospho rylation
Kras/ox cells
B
RASless cells
IMP
RAF[inactive]
14-3-3
low KSR[inactive]
MEK[inactive]
C
KSR expressing RASless cells
Ectopic KSR[active]
IMP
14-3-3
KSR[inactive]
MEK[inactive]
RAF[inactive]
Ectopic KSR[active]
RAF[active]
P
MEK[active]
MEK[inactive]
Allosteric activation
Phospho rylation

Figure S6. **Model of KSR-driven proliferation in RASless cells**. (A) Under normal conditions, active RAS recruits RAF kinases to the membrane and thereby relieves RAF kinases from their inhibitory closed confirmation. At the same time, IMP is also recruited to RAS which promotes its autoubiquitination, thereby facilitating membrane translocation of KSR and dimerization of KSR with RAF. Then, KSR allosterically activates the RAF kinases which in turn phosphorylate and activate MEK. (B) In Rasless cells, RAF remains in its inactive closed conformation which resides in the cytoplasm. KSR is also kept in an inactive complex $KSR_{inactive}$, light blue) with 14-3-3 and IMP proteins. (C) When KSR is present at high levels in RASless cells, some KSR molecules (KSR_{active} dark blue) may escape 14-3-3 and IMP-mediated inhibition and translocate to the membrane, where they recruit RAF and promote formation of its active conformation. RAF becomes fully active by dimerization with KSR, a process that requires active ATP binding. Active RAF then phosphorylates MEK and activates the MAPK pathway.
